# A Novel Motor Structure with Extended Particle Swarm Optimization for Space Robot Control

**DOI:** 10.3390/s23084126

**Published:** 2023-04-20

**Authors:** Hongwei Gao, Zide Liu, Xuna Wang, Dongyu Li, Tian Zhang, Jiahui Yu, Jianbin Wang

**Affiliations:** 1 School of Automation and Electrical Engineering, Shenyang Ligong University, Shenyang 110000, China; 2 Beijing Bolean Intelligence Technology Co., Ltd., Beijing 100000, China; zzddddddz@163.com; 3 Department of Biomedical Engineering, Zhejiang University, Hangzhou 310027, China; jiahui.yu@zju.edu.cn; 4 Ocean College, Zhejiang University, Hangzhou 316021, China; 5 China Telecom Zhejiang Branch, Hangzhou 310014, China

**Keywords:** bearingless switched reluctance motor, finite element analysis, particle swarm optimization, self-starting, step rotor

## Abstract

This paper studies motor structures and optimization methods for space robots, proposing an optimized stepped rotor bearingless switched reluctance motor (BLSRM) to solve the poor self-starting ability and significant torque fluctuation issues in traditional BLSRMs. Firstly, the advantages and disadvantages of the 12/14 hybrid stator pole type BLSRM were analyzed, and a stepped rotor BLSRM structure was designed. Secondly, the particle swarm optimization (PSO) algorithm was improved and combined with finite element analysis for motor structure parameter optimization. Subsequently, a performance analysis of the original and new motors was conducted using finite element analysis software, and the results showed that the stepped rotor BLSRM had an improved self-starting ability and significantly reduced torque fluctuation, verifying the effectiveness of the proposed motor structure and optimization method.

## 1. Introduction

With the rapid development of modern industry and the leap in aerospace technology, aerospace tasks are becoming more complex. At the same time, it is becoming more and more common for artificial satellites to fail due to fuel exhaustion. Satellites can fail because of a single component, and the costs are high. Therefore, countries are paying more and more attention to on-orbit service technology [1,2,3,4,5]. On-orbit service refers to providing services for human-crewed or uncrewed spacecraft in space, including the repair and replacement of spacecraft components, spacecraft relocation, and liquid transfer. In the complex and harsh environment of space, space robots can assist astronauts in completing complex missions and even replace astronauts in conducting some dangerous extravehicular operations. Space robots can assist in completing space operations and ensure the safety of astronauts and the successful completion of missions. Therefore, the use of space robots for on-orbit services [6] has attracted more and more attention and is becoming one of the main trends in the development of aerospace technology.

**Figure 1:**
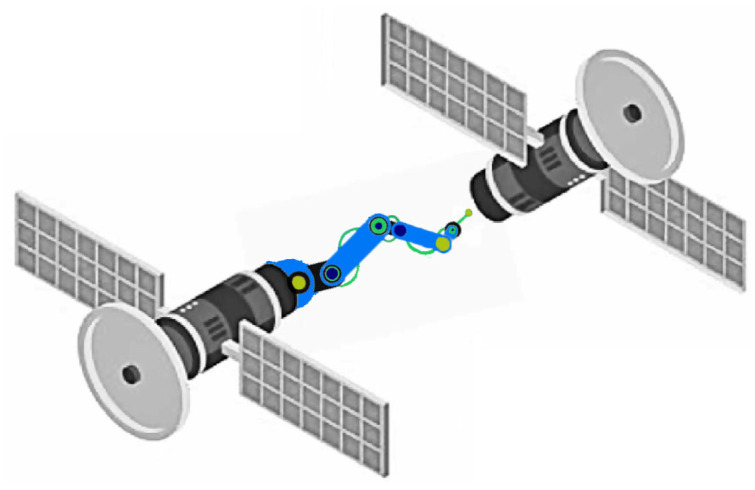
Concept image of a robotic arm for on-orbit service. The robotic arm is used to assist in the combination of the two satellites.

Space robots [7,8] providing extravehicular services can complete extensive tasks, including capturing and maintaining small satellites, target handling, and on-orbit assembly. The robots also put forward higher requirements for the performance of motor drives, and researchers are paying more and more attention to small, lightweight motors with high power density. The increase in motor speed not only helps to increase the power density of the motor, but also reduces the size and weight of the equipment, as shown in Figure 1. Therefore, high-speed and ultra-high-speed motors are increasingly being used in aerospace, flywheel energy storage, and other fields.

The bearingless switched reluctance motor (BLSRM) [9,10,11,12,13,14] is a new type of motor that combines drive and suspension. It combines bearingless technology and switched reluctance motors (SRM). SRM does not require permanent magnets or electromagnets, but instead uses the magnetic reluctance difference between the iron core and the stator windings to generate torque. The rotor and stator of the BLSRM are not supported by bearings, but by magnetic levitation technology. The bearingless motor has no mechanical wear and a long service life.

It has the advantages of having a compact structure, enormous output power, and no need for lubrication. In addition, it has the benefits of a simple structure and the robust fault tolerance of SRM. Compared with traditional servo motors, the BLSRM does not require vulnerable components such as brushes or slip rings, making it suitable for special requirements in the space environment, such as radiation resistance and high–low-temperature endurance. Its compact structure allows for a higher power output in the same space. The manufacturing cost is relatively low as it does not require a feedback control system or precise sensors. Its simple structure usually results in higher efficiency compared to servo motors, especially under partial load or low-speed operation. Due to its good fault tolerance, flexible control, and adaptability in harsh environments, it is widely used in aerospace.

However, due to the structure of the BLSRM motor itself, there are disadvantages, such as the significant torque fluctuation and existence of a torque dead zone. To solve these problems, many experts in the aerospace field and other areas have carried out research on the motor structure and performance. Many domestic and foreign universities and scientific research institutions have introduced bearingless switched reluctance motors [15,16,17,18,19,20]. Yue Qi proposed a novel hybrid rotor type 12/14 BSRM by keeping the air gap constant and modifying the aligned area between the torque and rotor poles to solve this problem. Fangxu Li changed a BLSRM with a 12/12 structure to reduce the axial space and produce higher torque for the purpose of improving performance. Zhenyao Xu proposed a stepped rotor structure and optimally designed the torque pole arc and rotor pole shape to solve the self-starting problems and reduce the torque ripple. Single-neuron-based rotor suspension control of a 12/14 hybrid pole BLSRM with autonomous rotation and suspension poles was presented. This hybrid pole structure produced suspension force, linearly concerning the rotor position. Wenjuan Hao proposed a bearingless linear switched reluctance motor to simplify the levitation force control based on a structural decoupling design for the mover. Shaofei Tang presented a 12/14 bearingless switched reluctance motor (BLSRM) with a small permanent magnet. However, due to the dead torque zone, BLSRM structures cannot self-start at certain rotor positions. 

Traditional motors, such as induction motors and permanent magnet synchronous motors, usually have rotors with iron cores. In contrast, the rotor of a BLSRM is coreless. Due to the absence of a core for support, when the rotor is in certain positions, the magnetic field on the rotor does not overlap with that on the stator, which makes it impossible to generate enough magnetic force to start the motor. Additionally, there are significant torque fluctuations during the start-up and acceleration processes. Therefore, we optimized the motor by focusing on two main aspects: we improved the stepped structure to eliminate the dead zone, and we solved the problem of the significant torque fluctuations during start-up.

The particle swarm algorithm [21,22] is also known as the bird swarm foraging algorithm or particle swarm optimization algorithm (particle swarm optimization, PSO). PSO has many advantages, such as a simple expression, easy implementation, few setting parameters, and a fast convergence speed. Since being proposed, it has attracted academic attention. At present, its application field has been extended from its initial function [23], neural network training [24], to practical engineering problems, such as optimal control [25,26] and structural optimization [27,28], and has achieved good results. In addition, PSO can handle multi-dimensional, nonlinear, and non-convex optimization problems, and is able to search for a global optimal solution. However, the problem of motor parameter optimization is usually a high-dimensional problem. In order to reduce torque ripple and improve the motor’s starting ability, in this paper, we consider torque pole arc, rotor pole arc, and step structure geometry parameters as optimization variables. In addition, PSO has been widely used in the design and optimization of motors. PSO is a heuristic optimization algorithm that optimizes problems by simulating the behavior of birds searching for food. However, the particle swarm optimization algorithm easily reverts to finding the local optimal solution, and is not suitable for high dimension problems..

In this study, we conducted the initial design and optimization of the BLSRM structure based on its structure and working principles. With the objective of addressing the issues of self-starting and oscillation by designing a stepped rotor, an optimization method for motor parameters was implemented by integrating finite element analysis with the PSO algorithm. The initial motor was optimized using this method to resolve the aforementioned problems. The main contributions of this paper are summarized as follows.

(1) Aiming to solve the problems of the poor self-starting ability and significant torque fluctuation of the 12/14 hybrid stator pole bearingless switched reluctance motor, we propose two critical parameters, namely, step height and step angle, and conducted an experimental analysis on the starting torque, average torque, and torque fluctuations under different conditions in order to design a stepped rotor bearingless switched reluctance motor structure. 

(2) To solve the problems of the need to rely on experience, the time- and labor-consuming nature, the high degree of specialization, and the difficulty of obtaining optimal the structural parameters of the current motor design, we combined the finite element analysis method and particle swarm algorithm to establish a systematic motor structure optimization method. 

(3) We improved the particle swarm optimization algorithm and enhanced its global optimization capability. Based on this method, we optimized the design of the stepped rotor bearingless switched reluctance motor. 

(4) The finite element analysis results demonstrated the following: Compared with the original motor, when the excitation current was 2A, the initial design of the stepped rotor bearingless switched reluctance motor increased by 0.11 N·min in the starting torque and decreased by 1.9% in torque ripple. After optimization, the torque ripple was further reduced by 9.2%. In contrast, the starting torque remained unchanged, proving that the stepped rotor bearingless switched reluctance motor structure was adequate and that the proposed motor optimization method was feasible.

The remainder of this paper is organized as follows: Section 1 reviews the related work of the BLSRM. In Section 2, we review the structure and working principle of the stepped rotor BLSRM and introduce the initial design proposal for the stepped rotor BLSRM. Section 3 introduces the standard particle swarm optimization algorithm (PSO) and the optimized particle swarm algorithm, considering the characteristics of the motor optimization problem. Section 4 combines the improved particle swarm algorithm with the finite element method to optimize the initially designed motor structure. Section 5 analyzes the motor’s performance using finite element analysis software to verify the motor structure’s effectiveness and compare it with the original 12/14 hybrid stator pole type BLSRM. Finally, Section 6 concludes the paper with a summary and discussion.

## 2. Motor Design for Space Robots

**Figure 2:**
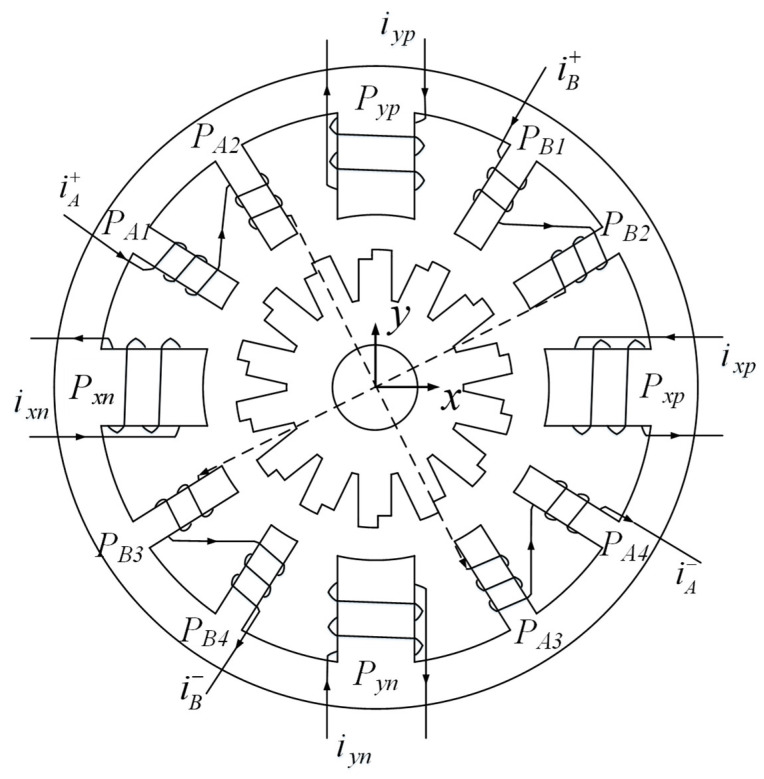
Structure of the stepped rotor BLSRM.

Due to the particularity of the working environment and tasks of space robots, there is a high requirement regarding the performance of the motor drive. Therefore, the design of the motor structure has a significant influence on the space robot. The traditional 12/14 hybrid stator magnetic pole BLSRM requires a better self-starting ability and significant torque fluctuation, which cannot directly meet the hardware requirements of space robots; thus, we redesigned the magnetic torque pole and rotor magnetic pole arc and adopted a stepped rotor based on its structure. As the number of poles of the stepped rotor increased during motor start-up, the magnetic field generated by the rotor was also gradually strengthened. Due to the gradual increase in the number of poles of the stepped rotor, the magnetic field of the rotor was able to interact more fully with the magnetic field of the stator, thereby improving the starting torque and torque stability of the motor. In this way, we designed a stepped rotor BLSRM structure, as shown in Figure 2.

### 2.1. Structure and Working Principle of the BLSRM

The rotor pole arc of the stepped rotor BLSRM was slightly larger than the torque pole arc so that, when the motor was started, no matter where the rotor was located, there would be a torque winding phase that would provide positive torque to the rotor.

In addition, the rotor’s stepped structure increased the motor inductance’s rising interval. According to the electromagnetic torque calculation, i.e., Formula (1), it was found that if the effective length of the torque winding conduction became more extensive, thereby increasing the torque during commutation, then the torque fluctuation would decrease.

(1) $$T=\frac{1}{2}i^{2}\frac{dL}{d\theta }$$

where T is the electromagnetic torque, i is the torque winding current, L is the inductance, and θ is the rotor position.

There are two types of poles, namely, suspension poles and torque poles, on the stator of a step rotor BLSRM. The stepped rotor BLSRM has two types of poles, a suspension force pole and a torque pole. The torque winding, composed of four coils in phases A and B, provides torque to rotate the rotor. $P_{xp}$,$\;P_{xn}$, $P_{yp}$, and $P_{yn}$ correspond to the positive and negative directions of the X-axis and the positive and negative directions of the Y-axis. We can satisfy the rotor’s requirement to be suspended by turning on one or two windings.

### 2.2. Parameter Conditions

**Figure 3:**
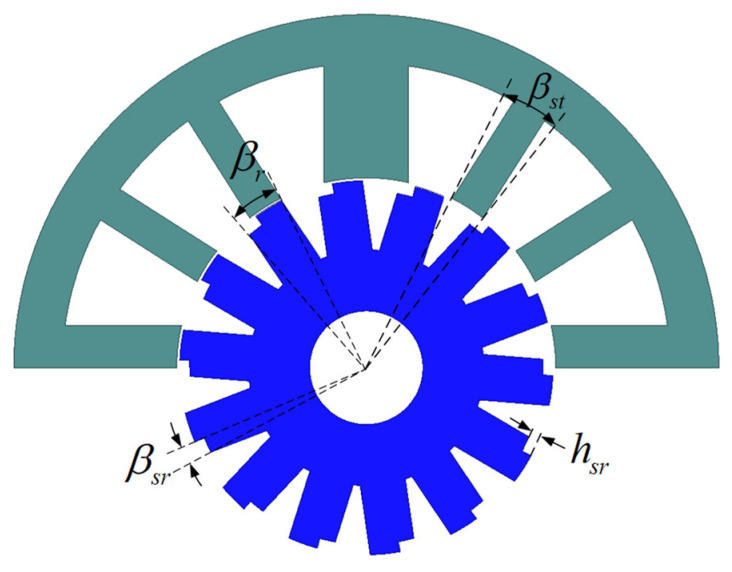
Important parameters of the stepped rotor BLSRM.

To compare the performance with the original 12/14 hybrid stator pole BLSRM and to verify the effectiveness of the stepped rotor BLSRM structure, we kept the main dimensions of the motor the same as those of the original motor, including the stator and rotor outer diameter, air gap length, and iron core length. In the design of the stepped rotor BLSRM, the rotor pole arc $\beta_{r}$ and the torque pole arc $\beta_{st}$ are critical parameters that directly affect the motor torque fluctuation and self-starting ability. Moreover, this paper has two unique and vital parameters: step height, $h_{sr},$ and step angle, $\beta_{rs}$, as shown in Figure 3.

To ensure that the motor has a specific self-starting ability at any rotor position, it is required that the rising periods of the inductance curves of the two adjacent phases have an inevitable overlap; that is, the rotor pole arc and torque pole arc are required to satisfy:

(2) $$\beta_{r}>\beta_{st}$$

**Figure 4:**
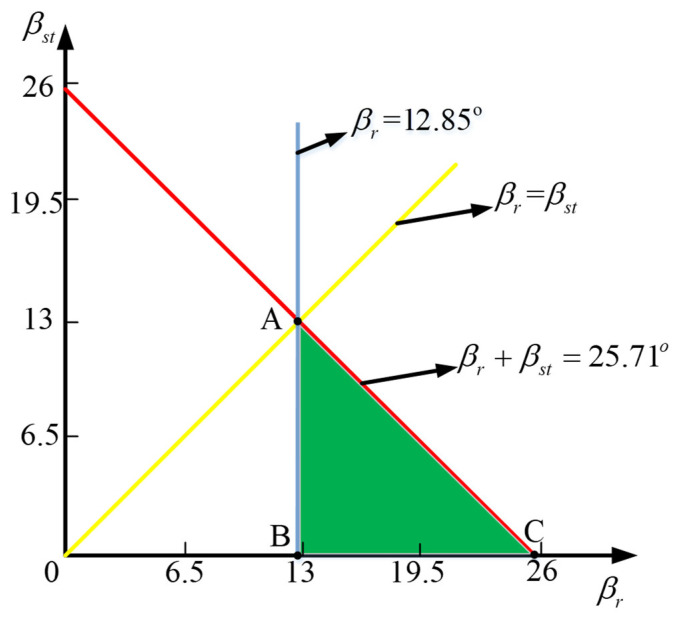
Feasible range of the rotor pole arc and torque pole arc.

To make the motor’s inductance as small as possible when the rotor is not aligned, and to generate positive torque at any rotor position, the rotor pole arc, $\beta_{r},$ and the torque pole arc, $\beta_{rs},$ must meet the following necessary conditions:

(3) $$\left\{\begin{array}{c} \beta_{r}+\beta_{st}\leq \frac{2\pi }{N_{r}}=\frac{360^{\circ }}{14}=25.7^{\circ }\\ \beta_{r}>\frac{2\pi }{qN_{r}}=\frac{360^{\circ }}{2\times 14}=12.85^{\circ } \end{array}\right.$$

where q is the number of phases of the motor and $N_{r}$ is the number of poles of the rotor pole. This can be expressed by the following triangle, ABC, in Figure 4.

The stepped structure has two parameters, namely, step angle, $\beta_{rs}$, and step height, $h_{rs}$. The design should be based on the principle of affecting other performance areas as little as possible to determine the range of variation, so $\beta_{rs}$ and $h_{rs}$ should meet the following conditions:

(4) $$\left\{\begin{array}{c} 1^{\circ }<\beta_{rs}<7^{\circ }\\ 0.1\;\mathrm{mm}<h_{rs}<0.5\;\mathrm{mm} \end{array}\right.$$

To ensure self-starting capability, the difference between the torque pole and rotor pole arc should be as slight as possible. According to the constraint relationship between the torque pole and rotor pole arc shown in Figure 4, and based on the design principle that the average torque of the motor should be as large as possible, we chose 12°/13.5°, 11.5°/14°, and 11°/14.5°. During the optimization of the motor structure, three combinations of uniformly distributed parameters of torque poles and rotor pole arcs were compared, and the average torque of one cycle was calculated.

#### 2.2.1. Design of Step Angle

The step angle is an essential parameter of the step rotor structure. It is a part of the rotor pole arc, and the stepped rotor structure can increase the rise interval of the motor inductance, allowing the motor to produce greater electromagnetic torque during start-up as well as low-speed operation, thereby improving the motor’s output torque. According to the relationship between the torque pole fox and the rotor pole arc, the design of the step angle can be divided into the following situations:

(5) $$\left\{\begin{array}{c} \;\beta_{rs}>\beta_{r}-\beta_{st}\\ \;\beta_{rs}=\beta_{r}-\beta_{st}\\ \;\beta_{rs}<\beta_{r}-\beta_{st} \end{array}\right.$$

According to these three situations and the feasible range of the rotor pole arc and torque pole arc shown in Figure 4, based on the determination of the main dimensions of the motor, the step height was fixed at 0.2 mm. The torque was not changed under different conditions. 

**Figure 5:**
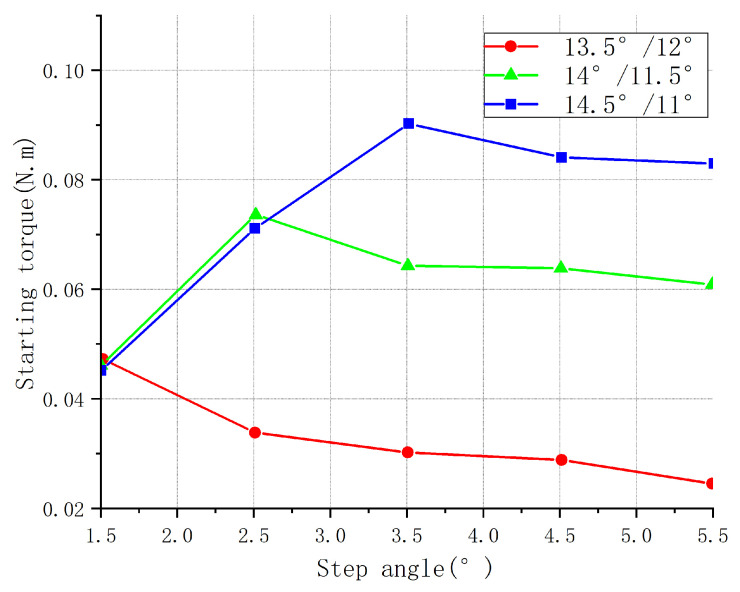
Starting torque of different step angles for three parameter combinations.

**Figure 6:**
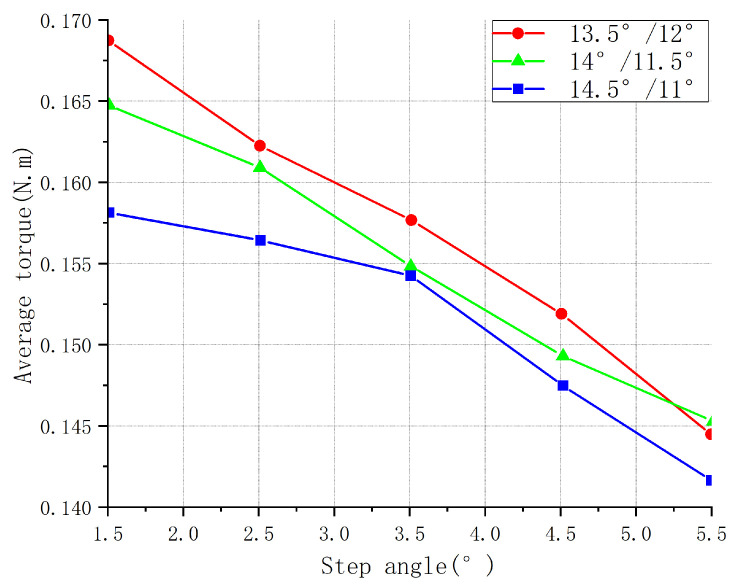
Average torque of different step angles for three parameter combinations.

**Figure 7:**
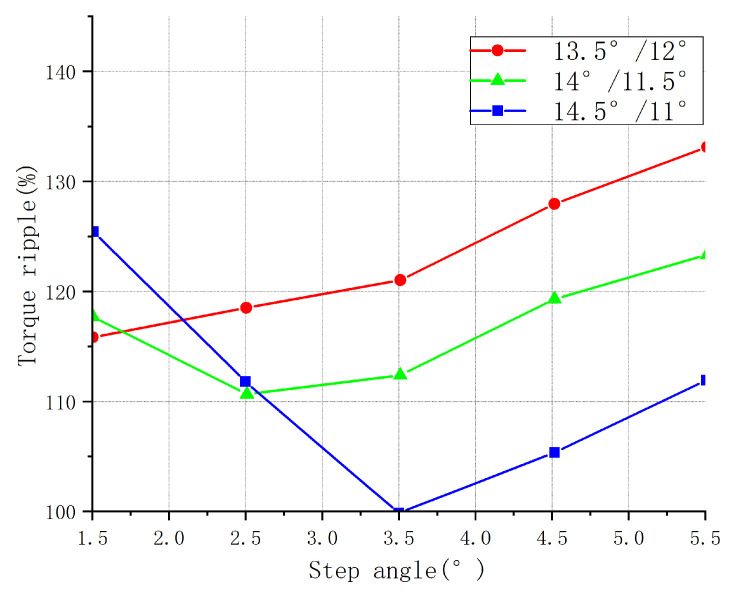
Torque fluctuation of different step angles for three parameter combinations.

Finite element analysis of the magnetic field was carried out for the combination of the three step angles of the pole and rotor pole arc. The average torque, starting torque, and torque fluctuation were calculated for the different step angles. Figure 5, Figure 6 and Figure 7 show the different torque characteristic curves for different step angles.

As can be seen in Figure 5, when the step angle was less than ($\beta_{r}-\beta_{st}$), the starting torque was proportional to the step angle; when the step angle was greater than ($\beta_{r}-\beta_{st}$), the starting torque was inversely proportional to the step angle. Regardless of the torque and rotor pole arc, the motor’s starting torque was the largest when the step angle was equal to ($\beta_{r}-\beta_{st}$).

The larger the step angle, the larger the angle of each step taken by the motor, and the fewer pulses required for each step. Therefore, the motor’s rotation speed increased in the same amount of time, but the torque output for each step decreased. From the average torque curve shown in Figure 6, the average torque decreased with the increase in the step angle.

The torque ripple curve shown in Figure 7 demonstrates that the torque ripple also adhered to the following laws with the increase in the step angle. When the number of magnetic poles was fixed, and if the stepping angle of the rotor was small, the magnetic field change of the motor was relatively smooth, so the torque fluctuation of the motor was small. When the stepping angle was large, the magnetic field change of the motor was relatively abrupt, possibly causing a larger torque fluctuation in the motor. When the step angle was less than ($\beta_{r}-\beta_{st}$), the torque ripple was inversely proportional to the step angle; when the angle was greater than ($\beta_{r}-\beta_{st}$), the torque ripple was proportional to the step angle.

To summarize, the starting torque and torque fluctuation were optimal when the step angle was equal to the difference between the rotor pole arc and the torque pole arc, so the size of the step angle was determined by the torque and the pole–pole arc of the rotor. That is,

(6) $$\beta_{rs}=\beta_{r}-\beta_{st}$$

#### 2.2.2. Design of Step Height

The direct effect of the stepped structure was to increase the air gap between the rotor pole and the torque pole. This reduced the intensity of eddy currents, decreased energy losses, and improved motor efficiency. If the step angle determined the range of the stepped structure, then the step height determined the strength of its effect. The proper step height was able to improve the positioning accuracy of the motor and reduce the probability of step loss occurring. At the same time, it also reduced the noise and vibration of the motor. When the torque winding current was constant, the motor torque was determined by the rate of change in the inductance. The step height directly affected the change in inductance, thus affecting the torque characteristics. Therefore, the torque characteristics were analyzed at different step heights.

**Figure 8:**
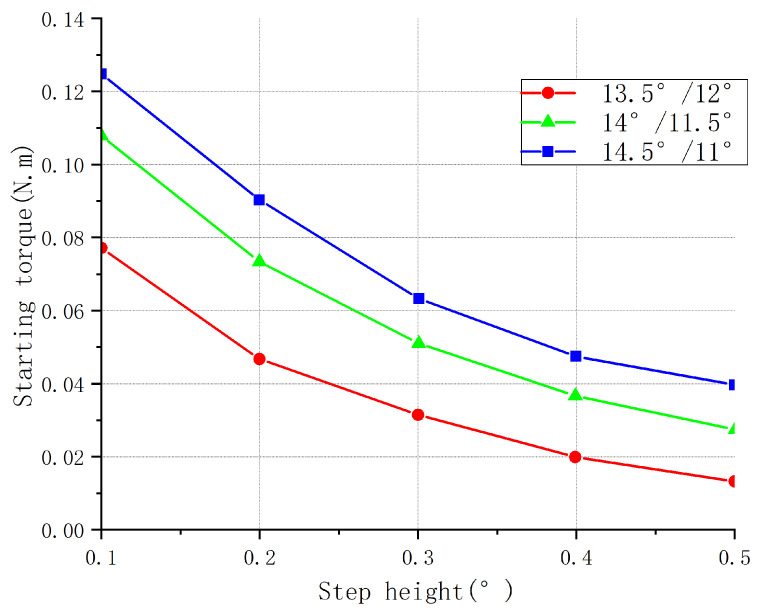
Starting torque of different step heights for three parameter combinations.

Figure 8 shows the starting torque curves for the structures of the three parameter combinations at different step heights. As the height of the stepped rotor increased, the inductance of the motor increased, as did the rise time of the inductance. This led to a decrease in the response speed of the motor, which, in turn, led to a slower generation of electromagnetic torque and a decrease in the starting torque of the motor. It can be seen in the figure that with the increase in the step height, the starting torque of the three structures gradually decreased. The starting torque of the torque pole arc–rotor pole arc was 11°/14.5° as a whole, larger than that of the other two structures: 11.5°/14° took second place, and 12°/13.5° was the smallest.

**Figure 9:**
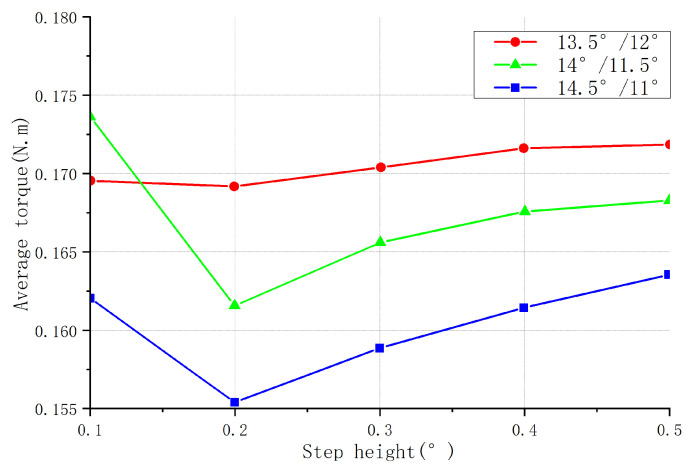
Average torque of different step heights for three parameter combinations.

Figure 9 shows the average torque curves for the structures of the three parameter combinations at different step heights. When the step height of the salient rotor of the stepped motor increased, the starting torque of the motor decreased, but the motor’s average torque was also affected. When the step height was relatively small, the motor’s torque curve was smaller, resulting in a higher starting torque, but a lower average torque. However, when the step height increased, the torque curve increased, resulting in a lower starting torque, but a higher average torque. According to the graph, the optimal performance was achieved when the step height was 14/11.5, with a step size of 0.1.

**Figure 10:**
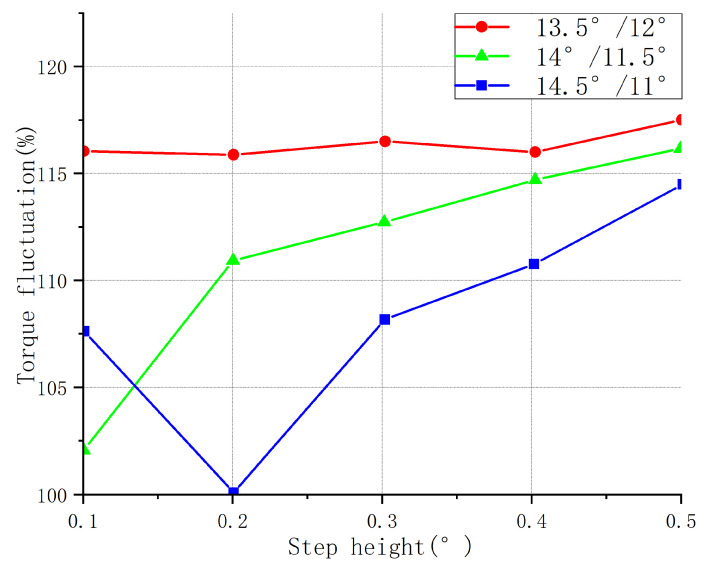
Torque fluctuation at different step heights for three parameter combinations.

Figure 10 shows the torque fluctuation curves for the three structures at different step heights. When the step height of the salient pole rotor increased, the required magnetic flux change for each step also increased accordingly. This led to a more uniform distribution of magnetic flux between the rotor and stator, reducing the rapid changes in magnetic flux and thus decreasing the torque ripple. This effect was more pronounced when the step height increased to an appropriate level. Figure 8, Figure 9 and Figure 10 show that when the rotor pole arc–torque pole arc was 13.5°/12°, the average torque of the motor was higher than that of the other two combinations. Its starting torque was higher than that of the two other combinations, and the torque ripple was more prominent, completely deviating from the original design intention of increasing the starting torque and reducing the torque ripple; thus, this combination was unsuitable. The 14°/11.50 and 14.5/11° structures had two rotor pole arc–torque pole arc combinations, each with 5 step heights, for a total of 10 combinations. The rotor pole arc and torque pole arc were visible. When the step height was 14°, 11.5°, or 0.1, the starting torque was the most significant, the torque fluctuation was the slightest, and the average torque was the highest.

In summary, the rotor pole arc, torque pole arc, step angle, and step height of the motor were determined as follows:

(7) $$\left\{\begin{array}{c} \beta_{r}=14{}^{\circ}\\ \;\;\;\beta_{st}=11.5{}^{\circ}\\ \;\beta_{rs}=2.5{}^{\circ}\\ \;\;\;\;\;\;\;h_{rs}=0.1\;\mathrm{mm} \end{array}\right.$$

According to the motor drive requirements of the space robot, based on the above analysis, we redesigned the motor structure of the stepped rotor BLSRM, thereby improving the performance of the space robot. Then, we optimized the PSO algorithm and used finite element electromagnetic field analysis software to determine the structural parameters that significantly affected the torque and the structural parameters of the step angle on the rotor. Moreover, we assessed the performance of the motor and further completed the preliminary design of the stepped rotor BLSRM structure.

## 3. Improved PSO

In PSO, the n-dimensional problem to be optimized corresponds to an n-dimensional solution space. The potential solution for the problem to be optimized is regarded as the mass-free and volume-free moving particles in that space, described by the position and velocity such that each particle has different characteristics, thus forming a particle population. During PSO, an evolutionary search is carried out through the cooperation and competition of each particle in the population.

PSO achieves the purpose of optimization by completing an evolutionary search through the cooperation and competition of each particle in the group. The key to this algorithm is to update the particle position and velocity, that is, to approach the optimal global solution quickly. Each particle updates itself by tracking the optimal solutions for individual and group extreme values. For the optimal particle of the group, the powerful personal matter and the extreme group value are relevant. Therefore, when the particle is updated, the learning ability of the self-cognition and social cognition parts is zero, and there is no autonomous evolution mechanism. Therefore, it is very likely that it will fall into local extreme points and be unable to escape, so the PSO algorithm can mature quickly and early.

We proposed eliminating the inferior and bolstering the superior value to solve this problem. The main idea is that during iterative updates, we sort the entire particle swarm according to the fitness value and eliminate the $2^{n}$ particles with lower fitness values in this generation, according to the dimension, n, of the problem. Taking the particle with the most significant group fitness value as the center, we divide the solution space into $2^{n}$ regions, and $2^{n}$ new particles with different directions and uniform distributions are generated in these new regions. 

When there is a new particle whose fitness value is larger than that of the original optimal particle of the group, the group extremum is updated; that is, it is successful in helping the group’s optimal particles to escape the optimal local solution. The key to PSO is updating the particle position and speed, that is, quickly growing close to the optimal global solution. This paper addresses the problems of the standard PSO and combines them with the optimization of the structural parameters of the motor to improve the standard PSO.

In this paper, the adaptive inertial weight method is adopted; that is, the inertial weight changes with the change in the particle’s fitness value. The expression is as follows:

(8) $$\omega =\left\{\begin{array}{c} \omega_{min}+\frac{\left(\omega_{max}\;-\;\omega_{min}\right)\;*\;\left(f\;-\;f_{min}\right)}{\left(f_{avg}\;-\;f_{min}\right)},f\leq f_{avg}\\ \omega_{max},f>f_{avg} \end{array}\right.$$

where f represents the current fitness value of the particle, $f_{min}$ and $\;f_{avg}$ represent the minimum fitness value and the average fitness value of all the current particles, and $\omega_{max}$ and $\omega_{min}$ represent the maximum and minimum values, respectively, usually taken as $\omega_{max}=0.9$ and $\omega_{min}=0.4$.

The learning factor $c_{1}$ decreases with the increase in the number of iterations, and $c_{2}$ increases with the increase in the number of iterations, meeting the requirements, and generally follows $c_{1}+c_{2}\leq 4$. The change formula of the learning factor is

(9) $$\left\{\begin{array}{c} c_{1}=c_{1,ini}+\left(c_{1,fin}-c_{1,ini}\right)\frac{t}{t_{max}}\\ c_{2}=c_{2,ini}+\left(c_{2,fin}-c_{2,ini}\right)\frac{t}{t_{max}} \end{array}\right.$$

where $c_{1,ini}$ and $c_{2,ini}$, respectively, represent the initial values of $c_{1}$ and $\;c_{2}$, and $c_{1,fin}$ and $\;c_{2,fin}$, respectively, represent the final values of $c_{1}$ and $c_{2}$. In this paper, $c_{1,ini}=2.5$, $c_{2,ini}=0.5$, $c_{1,fin}=0.5$, and $c_{2,fin}=2.5$.

**Figure 11:**
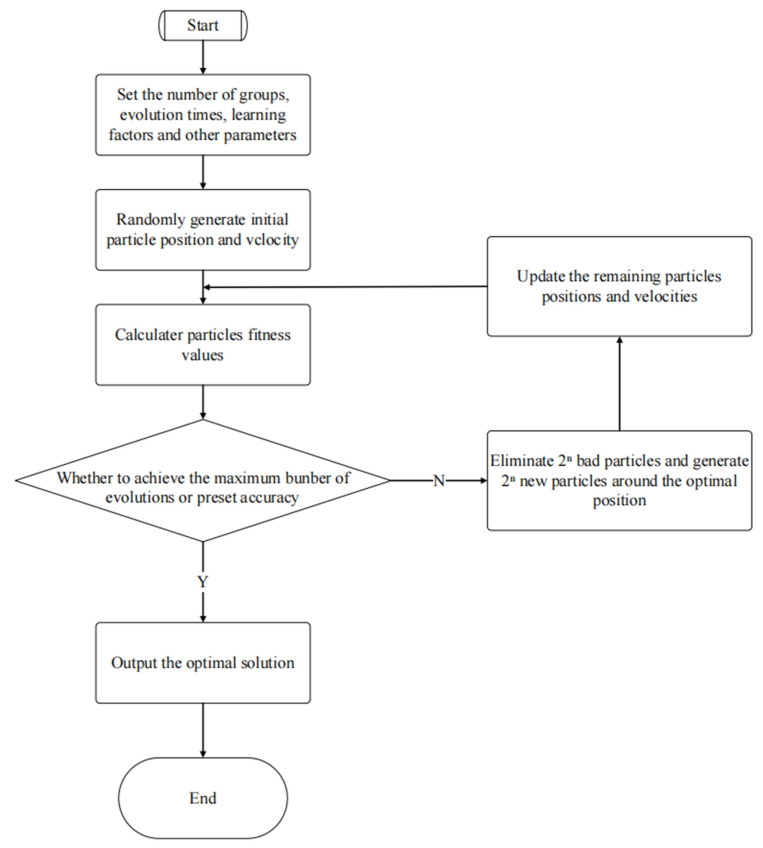
Improved PSO flow chart, showing the basic process of the improved PSO.

The formula for generating new particles is as follows:

(10) $$\left\{\begin{array}{c} V_{i}\left(t\right)=\left(1-t/t_{max}{\left(\right)}_{d,l}{}_{gbest}\right)\\ X_{i}\left(t\right)=X_{gbest} \end{array}\right.$$

where t and $t_{max}$ are the current number of iterations and the maximum number of iterations, respectively, and $1-t/t_{max}$ is a coefficient that linearly decreases as the number of iterations increases. It can make the optimal particle search step of the group more prominent and increase the probability of escaping from the local optimum in the early stage of optimization, as well as making the search step size of the optimal particle of the group smaller in the later stage of optimization, which is beneficial to improving the accuracy of the algorithm. $V_{gbest}$ and $X_{gbest}$, respectively, are the speed and position of the optimal particle of the group, and $Direc_{t,l}$ is the direction of the first particle in the first cutting area, where d,l meet the following conditions:

(11) $$\left\{\begin{array}{c} 2\leq d\leq 2^{n}\\ 1\leq l\leq ESC \end{array}\right.$$

where *ESC* is the escape capability coefficient, which represents the number of new particles generated in each direction, meaning that the *ESC* is an integer greater than or equal to 1. In dealing with a complex multipolar value problem, there is a large difference in fitness value between the optimal local value and the optimal global value, which produces a new particle in each direction; thus, the escape ability coefficient *ESC*, which represents the number of new particles generated in each direction, is set as an integer greater than or equal to 1. When it cannot escape from the local optima, the coefficient can be appropriately increased to further enhance the global search capability of the algorithm. We should ensure that Formula (10) is satisfied when adjusting the escape ability coefficient. Figure 11 shows the Improved PSO flow chart.

(12) $$\left\{\begin{array}{c} ESC\geq 1\\ 2^{n}\cdot ESC<sizepop \end{array}\right.$$

It can be seen that the elimination of the particles with lower fitness values and the procedure of generating new particles are merged into the update iterative process, so the algorithm steps do not become complicated, and the algorithm can help the particles to escape from the optimal local domain while eliminating particles with relatively poor fitness values. The particles also improve the global convergence speed of the algorithm.

This section described the process of improving the particle swarm optimization algorithm and enhancing its global optimization ability. Next, to solve the common problems of the current motor design, such as the need to rely on experience, the time-consuming and labor-intensive nature, the high degree of specialization, and difficulty of obtaining optimal structural parameters, the finite element analysis method and particle swarm algorithm were combined to establish a systematic motor structure optimization. The technique was used to optimize the stepped rotor bearingless switched reluctance motor design.

## 4. Optimized Design of Stepped Rotor BLSRM

### 4.1. The Selection of Optimization Variables

To reduce the torque fluctuation, improve the motor’s starting ability, and ensure the motor’s output torque, the torque pole $\mathrm{arc}\;\beta_{st}$ of the motor, the rotor pole $\mathrm{arc}\;\beta_{r}$, and stepped structure geometry parameters were considered as optimization variables first. However, as $\beta_{rs}$ was part of the rotor pole $\beta_{r}$, the step angle was interrelated and could not be used as an optimization variable simultaneously. To maximize the effect of the stepped structure, we chose the torque pole $\mathrm{arc}\;\beta_{st}$, rotor pole $\mathrm{arc}\;\beta_{r}$, and step height $h_{rs}\;$ as the optimization variables. The step angle should satisfy the following condition:

(13) $$\beta_{rs}=\beta_{r}-\beta_{st}$$

### 4.2. Constraint Setting

To ensure that the motor has a specific self-starting ability when the torque winding current is constant at 2A, the starting torque $T_{s}$ met the following condition:

(14) $$T_{s}\geq 0.05\;N\cdot m$$

Increasing the motor’s starting torque risked causing the output torque to decrease. Therefore, during the optimization process, the average torque $T_{ave}$ of the motor during a running cycle met the following condition:

(15) $$T_{ave}\geq 0.9T_{ave0}$$

where $T_{ave0}$ is the average torque of the original 12/14 BLSRM in an operating cycle.

### 4.3. Determination of the Objective Function

To solve the problems of the low starting torque and significant torque fluctuation of the 12/14 BLSRM, the starting torque of the motor was restrained by Formula (12) to enhance the self-starting ability. The average torque was calculated using Formula (13) to guarantee the output torque of the motor. Therefore, selecting torque fluctuation as the only optimization target was a necessary step, ensuring the optimization effect and simplifying the optimization problem.

The objective function is defined as

(16) $$F\left(x\right)=T_{ripple}$$

The calculation of the torque fluctuation was as follows:

(17) $$T_{ripple}=\frac{T_{max}-T_{min}}{T_{ave}}\times 100\%$$

### 4.4. Method for Calculating Fitness Value Based on Finite Element

The accuracy of the optimization target calculation is paramount when using PSO to solve practical problems. However, BLSRM is a complex system with nonlinear, strong coupling. When deriving its mathematical model, it is easy to ignore edge effects, magnetic saturation, and magnetic leakage. Moreover, the rotor’s particular stepped structure complicates this paper’s mathematical model. Therefore, an optimization target calculation method based on finite element electromagnetic field analysis is more accurate than a calculation method based on mathematical models.

**Figure 12:**
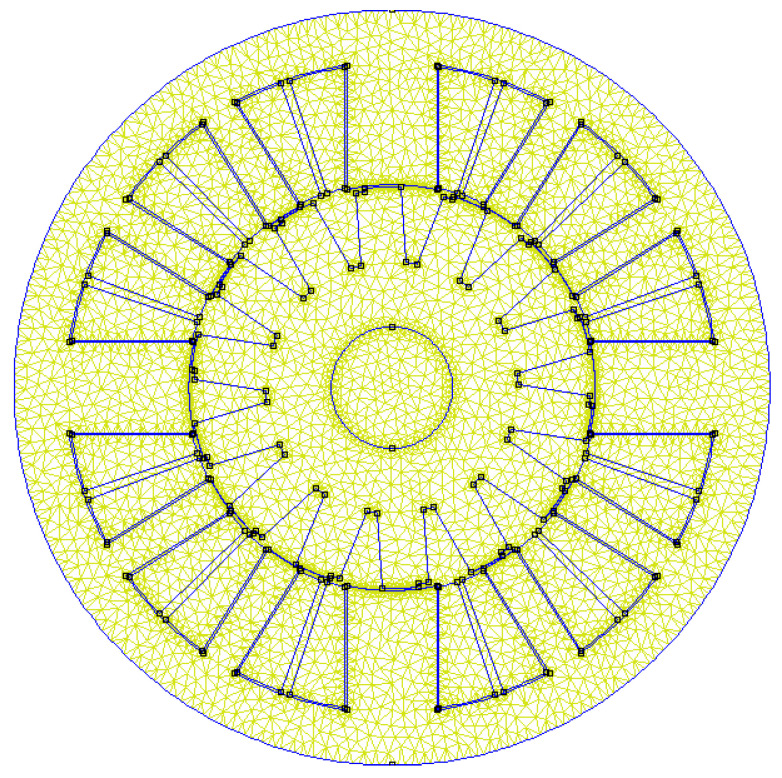
Finite element model of the stepped rotor BLSRM.

The fitness function was used to evaluate the fitness of each particle. The fitness function was designed based on the objective function and torque ripple, and its purpose was to map the value of the objective function to the fitness value for comparison and selection by the optimization algorithm. Generally, the smaller the value of the fitness function, the more suitable the particle is for the optimization problem. We used the FEMM 2D electromagnetic field analysis software to model and analyze each particle. First, we established the finite element model, shown in Figure 12, according to the position information of the particle. Then, we carried out an analysis to obtain the maximum, minimum, and average torques. Following this, we calculated the torque ripple, that is, the fitness value.

### 4.5. Algorithm Implementation

**Figure 13:**
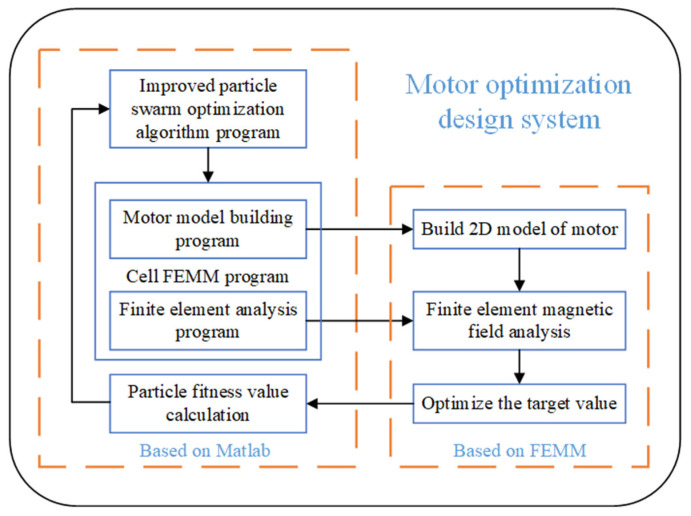
Block diagram of the stepped rotor BLSRM optimization design system.

Figure 13 shows the block diagram of the optimized design system of the stepped rotor BLSRM.

We used Matlab to prepare the improved particle swarm algorithm program for the optimization design process. We connected it to the 2D finite element analysis software FEMM. We repeatedly called the FEMM software to complete the model building and electromagnetic field analysis of the finite element of each particle in the optimization process. Moreover, we obtained performance indicators such as the starting torque, average torque, and torque fluctuation to calculate the fitness value of each particle.

The optimization process was as follows:

Optimization process

Begin

Step 1: Set the parameters such as the inertia weight, learning factor, and escape ability coefficient;

Step 2: Randomly generate an initial group;

Step 3: Call the FEMM finite element magnetic field analysis software to model and analyze each particle and return the optimization target value;

Step 4: Calculate the fitness value of the particle according to the returned optimization target value;

Step 5: Compare the initial individual extreme importance and the initial group extreme value after calculating the fitness values of all the particles in the group;

Step 6: Perform iteration, sort the entire particle swarm by fitness value, eliminate the $2^{n}$ particles with the lower fitness values in the generation according to the number of variables, generate new $2^{n}$ particles at the particle positions with the maximum fitness values, and give them different directions and uniformly distributed speeds;

Step 7: Update the positions and velocities of the remaining particles;

Step 8: Update the individual extremum values and the group extremum values;

Step 9: If the stop condition is met (the preset accuracy or the number of iterations is reached), stop the search, and the result will be the output; otherwise, return to step 6 to continue the search.

End

**Figure 14:**
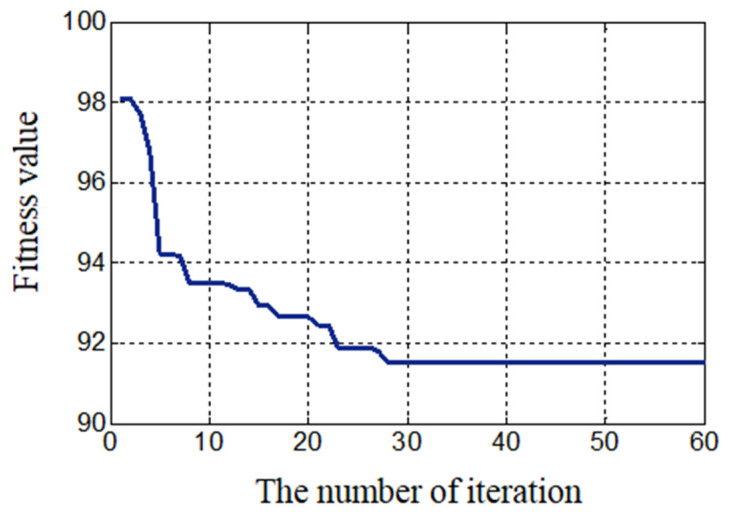
Convergence curve of the objective function.

Figure 14 shows the change curve of the fitness value of the optimal particle in the stepped rotor BLSRM parameter optimization process as the number of iterations increased. It can be seen that the most optimal solution was found when the 28th iteration was reached.

## 5. Analysis and Verification of Results

To improve the motor drive performance of the space robots, we used finite element electromagnetic field analysis software to analyze various aspects of the stepped rotor BLSRM in order to verify the effectiveness of the improved PSO algorithm and the motor structure.

### 5.1. Optimization Algorithm Test

**Figure 15:**
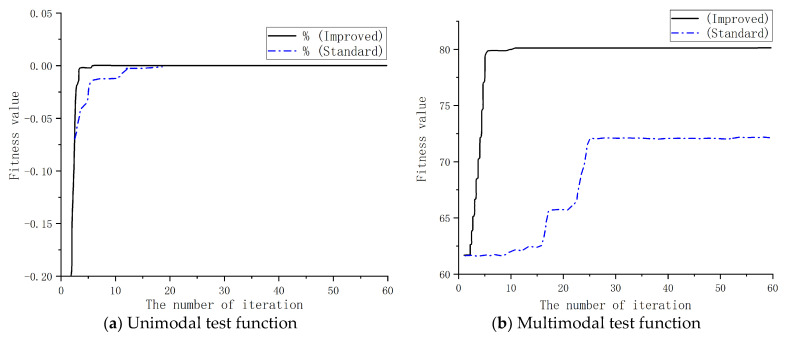
Comparison of convergence processes.

To verify the effectiveness of the improved PSO, single-peak and multi-peak test functions were used to test and compare the PSO before and after the improvement. Single-peak test functions are used to evaluate the global search ability and convergence speed of optimization algorithms, and they are a type of function with only one extreme point. Multi-peak test functions, on the other hand, contain multiple optimal solutions and are more suitable for comparing the global search abilities of two algorithms. Figure 15 compares the convergence process of the objective functions of the two algorithms. Table 1 shows the comparison of the optimization results.

It can be seen in Figure 15a and Table 1 that both the improved PSO and the standard PSO converged to a position close to 0 under the unimodal test function. The improved PSO achieved the design requirements after 5 iterations, while the standard PSO reached them after about 20 iterations. Therefore, the improved PSO improved the search efficiency and accuracy of the algorithm. Under the multimodal test function of Figure 15b, it can be seen that the objective process of the standard PSO converged to a local optimal solution position after about 25 iterations, rather than the optimal global solution. In contrast, the optimization result of the improved PSO accurately converged to the optimal global position after five iterations. For multi-extreme optimization problems, the standard PSO was able to easily stay at the local optimal solution position, unable to escape, and was affected by the parameter settings, initial populations, and other factors. However, the improved PSO ran the local optimal solution position and searched for the optimal global solution. Through the improved PSO algorithm, the optimized values of the rotor pole arc, torque pole arc, step angle, and step height of the motor could be calculated.

### 5.2. Characteristic Analysis of BLSRM

We use finite element electromagnetic field analysis software to analyze various characteristics of the stepped rotor BLSRM.

#### 5.2.1. Inductance Characteristics

**Figure 16:**
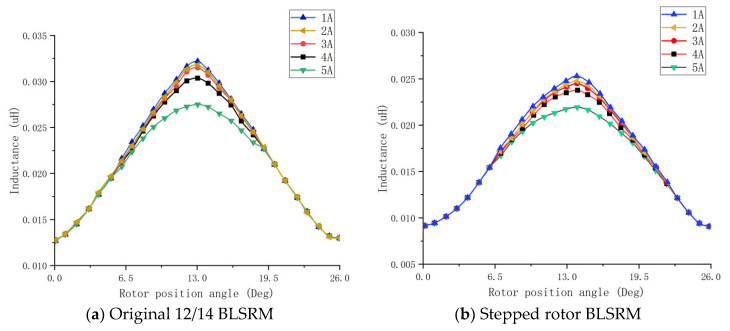
Inductance characteristic curve of torque windings.

Figure 16a,b show the torque winding inductance characteristic curves of the original 12/14-pole BLSRM and the stepped rotor BLSRM, respectively, at different currents. It can be seen in the figure that when the wind was fixed, the inductance characteristics of the two BLSRM torque windings were similar on the whole, and they both changed significantly with the change in the rotor position. The overlap area of the rotor poles was related, and the overlap area varied with the rotor position angle. From a local point of view, the inductance rise period of the stepped rotor-type BLSRM occupied a more extensive range; it did not remain at the maximum inductance position, but continued to change because the rotor pole arc was slightly larger than the torque pole arc.

#### 5.2.2. Torque Characteristics

**Figure 17:**
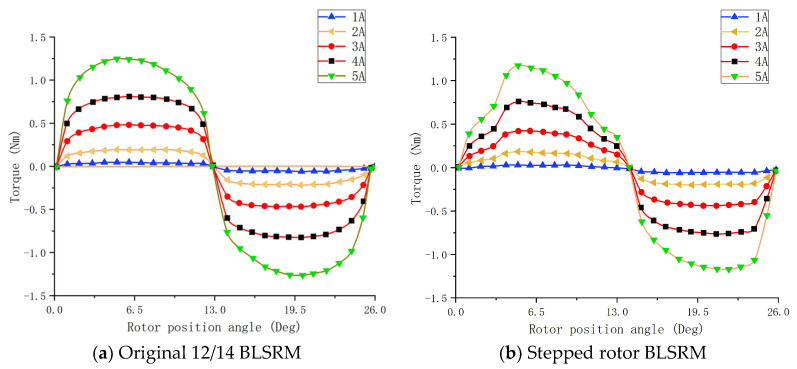
Torque characteristic curve of torque windings.

Figure 17 shows the torque characteristics of the torque windings at different currents for the original 12/14 pole type BLSRM and the stepped rotor BLSRM. According to the previous analysis of the inductance characteristics, the inductance varied with the rotor position angle. When the current is constant, the motor torque should be proportional to the rate of change in the inductance. It can be seen in the figure that the motor torque changed significantly with the change in the rotor position angle. The positive torque interval corresponded to the inductance rise phase, and the negative torque interval corresponded to the inductance fall phase. The stepped rotor BLSRM produced slightly less torque than the original BLSRM because the new motor had a more minor torque pole arc than the original, resulting in a slightly smaller inductance at the aligned position, and, thus, a slightly smaller slope in the inductance rise phase. However, since the inductance rise interval of the stepped rotor BLSRM was longer than that of the original BLSRM, the range of positive torque was more comprehensive, and the average torque generated by the new motor in one operating cycle did not decrease significantly.

#### 5.2.3. Starting Torque and Torque Ripple

**Figure 18:**
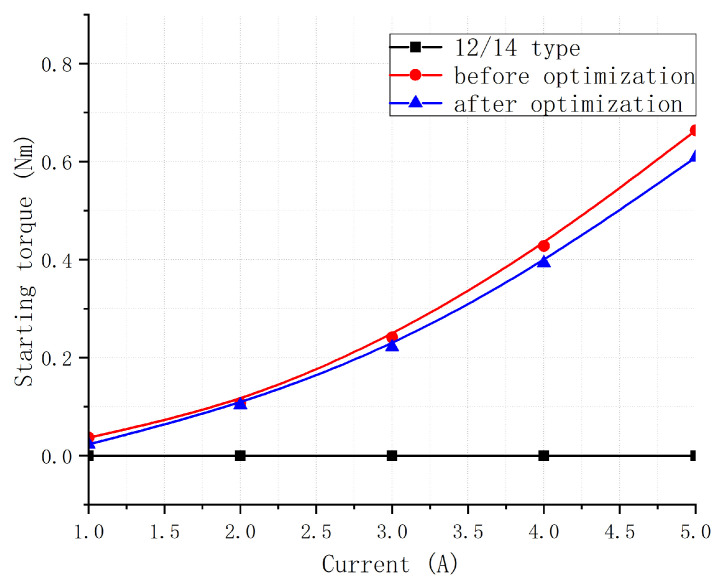
Comparison of starting torques of three motor structures.

**Figure 19:**
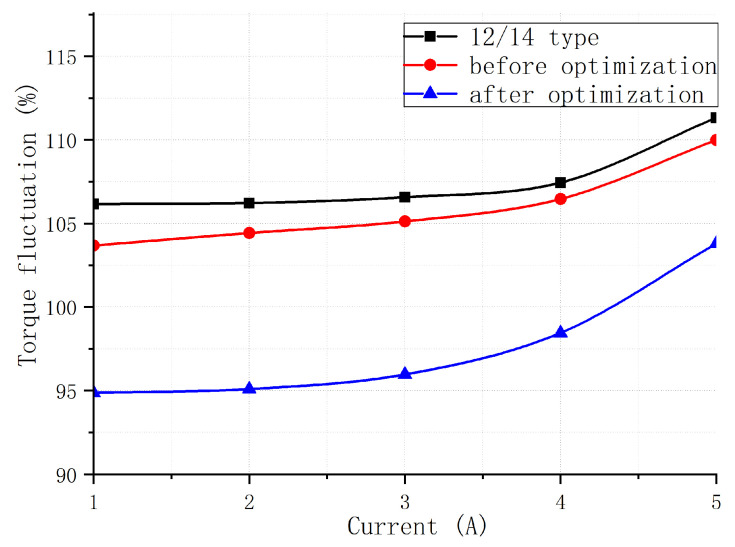
Comparison of torque fluctuations of three motor structures.

Figure 18 compares the original 12/14 type BLSRM with the stepped rotor BLSRM before and after optimization. Figure 19 shows a torque ripple comparison of the two. It can be seen that the starting torque was significantly improved compared to the 12/14 BLSRM due to the initial design of the stepped rotor BLSRM. Moreover, the torque ripple of the motor was significantly reduced, and the advantage of the starting torque remained. Compared with the original 12/14 type BLSRM, the optimized stepped rotor BLSRM had the ability to self-start, significantly reducing the torque fluctuation. Figure 18 shows that when the torque winding current was 1A, the starting torque of the new motor was slightly larger than that of the original motor. With the increase in the current, the starting torque advantage of the new motor became more pronounced, proving that the self-starting ability of the new motor was enhanced. Figure 19 shows that, in the case of different torque winding currents, the torque fluctuation of the improved motor was always significantly smaller than that of the original motor.

#### 5.2.4. Structural Parameter Comparison

Table 2 shows the optimization results, initial design, and structural parameters of the original 12/14 BLSRM and the stepped rotor BLSRM. It can be seen that the optimized motor rotor pole arc, torque pole arc, step angle, and step height are all different from the original values.

### 5.3. Dynamic Simulation

To further verify the effectiveness of the stepped rotor BLSRM structure, in this paper, we used Matlab to build a motor system simulation model and to perform dynamic simulations.

This section focuses on the simulation of the motor from start-up to the point at which a predetermined speed was reached, assuming that the rotor had been steadily suspended in the central position, that the initial torque load was 0.1 N.m, and that there was no load other than the gravity of the radial rotor and the shaft itself.

**Figure 20:**
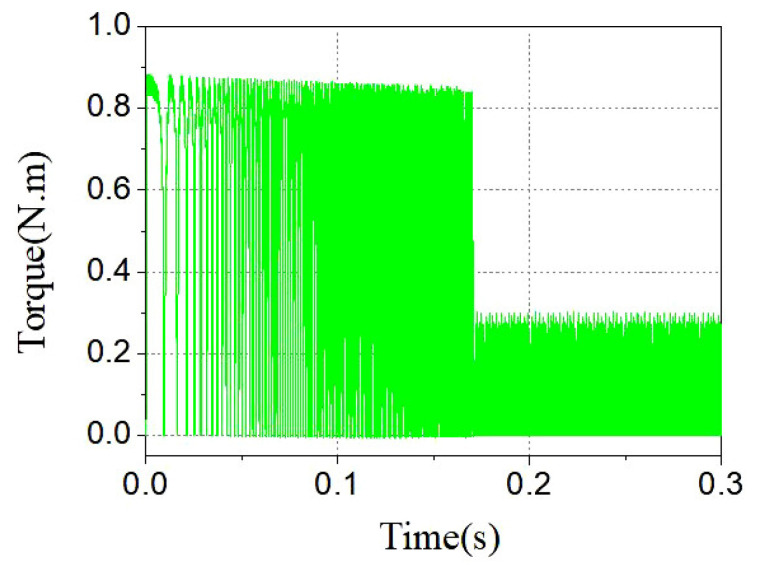
Torque variation during the speed regulation process.

**Figure 21:**
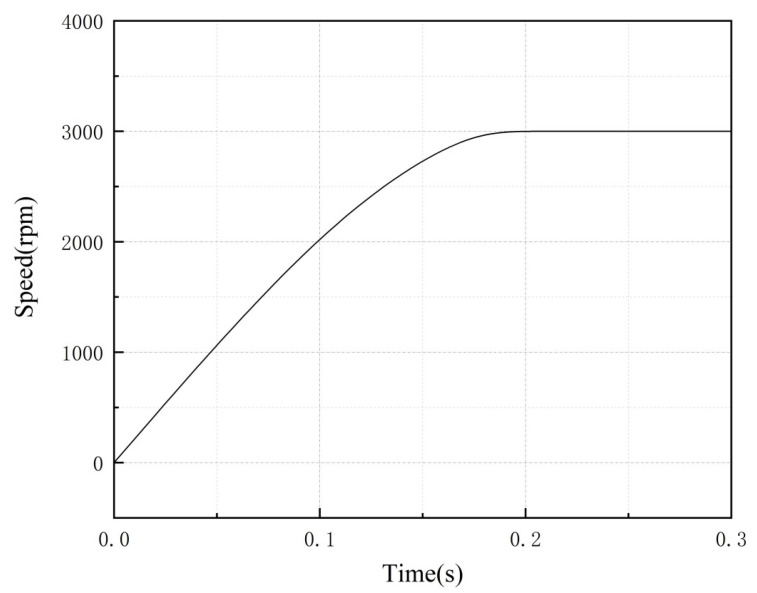
Speed variation during the speed regulation process.

Figure 20 and Figure 21 show the torque and speed variation curves. It can be seen that the alternating conduction of the two-phase torque windings of the A phase and the B phase were able to generate continuous torque to accelerate the rotor. During the starting stage, the torque ripple of the stepped rotor structure motor was small, and the speed increased steadily. After reaching the predetermined speed of 3000 rpm, the torque remained almost constant. This demonstrates that, under appropriate control strategies, the stepped rotor BLSRM structure improves its self-starting capability, has a larger starting torque value, and reduces torque ripple, showing a good dynamic performance. 

## 6. Conclusions

Firstly, aiming at aerospace tasks and considering the application of space robots, we designed a stepped rotor BLSRM structure to optimize the motor drive performance of a space robot based on the 12/14 hybrid stator pole type BLSRM. We also analyzed the influence of its motor structure and parameter conditions on the motor’s performance. Using an excitation current of 2 A, a torque pole arc of 11.5°, a rotor pole arc of 14°, a step angle of 0.1°, and a step height of 2.5 mm, the improved switched reluctance motor without bearings showed an increase of 0.11 N·m in starting torque and a reduction of 1.9% in torque ripple compared to the original motor design. Secondly, aiming to solve the prematurity problem of the standard PSO, we proposed a method by which the fittest particles would help the best to escape in order to improve the algorithm. According to the stepped rotor BLSRM structure characteristics, we combined the finite element method to calculate the fitness values and formed a motor structure optimization method based on the improved PSO. We used it to complete the optimization design of the stepped rotor BLSRM structure. After optimization, it was found that the torque pole arc was 10.3°, the rotor pole arc was 13.61°, the step angle was 0.22°, and the step height was 3.31 mm. Compared with the original initial motor, the torque ripple was further reduced by 9.2%. The starting torque remained unchanged. Finally, the effectiveness and feasibility of the optimization algorithm and motor structure were verified through algorithm testing and finite element analysis. The results show the following:

(1) Compared with the standard PSO, the proposed PSO had a faster convergence speed, higher accuracy, and more robust global optimization capability;
(2) Compared with the original 12/14 type BLSRM, the torque ripple of the stepped rotor type BLSRM w reduced, and the starting torque is significantly increased. Furthermore, the optimized motor reduces the torque ripple under the condition of ensuring self-starting ability. This proves that the stepped rotor BLSRM structure is adequate and feasible.

## Figures and Tables

**Table 1:** Comparison of PSO optimization results before and after improvement.

	Standard PSO	Improved PSO	The Optimal Value
Unimodal function	−3.43 × 10^−5^	−8.53 × 10^−26^	0
Multimodal function	72.64	80.70658	80.70658

**Table 2:** Structural parameters of stepped rotor BLSRM before and after optimization.

Parameter	Type 12/14	Stepped Type Initial Value	Stepped Type Optimization Value
Phase	2	2	2
Suspension force pole	4	4	4
Torque pole	8	8	8
Number of rotor poles	14	14	14
Suspension force pole arc (deg)	25.7	25.7	25.7
Torque pole arc (deg)	12.85	11.5	10.3
Rotor pole arc (deg)	12.85	14	13.61
Air gap length (mm)	0.3	0.3	0.3
Stator outer diameter (mm)	112	112	112
Stator yoke height (mm)	7.7	7.7	7.7
Rotor outer diameter (mm)	59.6	59.6	59.6
Rotor yoke height (mm)	9.7	9.7	9.7
Core length (mm)	40	40	40
Shaft diameter (mm)	18	18	18
Stepped angle (deg)	-	0.1	0.22
Stepped height (mm)	-	2.5	3.31

## Data Availability

The data used to support the findings of this study are available from the corresponding author upon request.

## Abbreviations

BLSRM	Bearingless Switched Reluctance Motor
SRM	Switched Reluctance Motors
PSO	Particle Swarm Optimization
$\beta_{r}$	Rotor Pole Arc
$\beta_{st}$	Torque Pole Arc
$h_{sr}$	Step Height
$\beta_{rs}$	Step Angle
